# Postoperative Pain Trajectories, Predictors of Severe Pain, and Satisfaction After Bariatric Surgery: A Prospective Multicenter Cohort Study

**DOI:** 10.7759/cureus.103542

**Published:** 2026-02-13

**Authors:** Salah N EL-Tallawy, Joseph V Pergolizzi, Abdullah T Alsubaie, Rania S Ahmed, Elsayed A Yousef, Khalid M Alsaeed, Haneen F Amlih, Hassan M Hetta, Tarek A Abdelzaher, Jumanna M Baaj, Mohamed S Nagiub, Issam S Shaheen, Radwa H Ahmed, Wegdan A Ali

**Affiliations:** 1 Anesthesia and Pain Management, King Abdulaziz and King Khalid University Hospitals, College of Medicine, King Saud University, Riyadh, SAU; 2 Anesthesia and Pain Management, Faculty of Medicine, Minia University, Minia, EGY; 3 Pain Management, National Cancer Institute (NCI) Cairo University, Giza, EGY; 4 Pain Management, NEMA Research Inc, Naples, USA; 5 Anesthesia and Pain Management, King Abdulaziz University Hospital, King Saud University Medical City, Riyadh, SAU; 6 Medicine, Alfaisal University College of Medicine, Riyadh, SAU; 7 Anesthesia and Pain Management, King Abdulaziz University Hospital, College of Medicine, King Saud University, Riyadh, SAU; 8 Anesthesia and Pain Management, King Khalid University Hospital, King Saud University Medical City, Riyadh, SAU; 9 Anesthesia, ICU, Pain Management, Faculty of Medicine, Minia University, Minia, EGY; 10 Medicine, College of Medicine, Badr University, Badr City, New Cairo, EGY; 11 Clinical Pathology Department, Faculty of Medicine, Assiut University, Assiut, EGY

**Keywords:** bariatric surgery, esp, fascial plane block, pain management, patient satisfaction, postoperative pain, predictors of severe postoperative pain, sleeve gastrectomy, tap

## Abstract

Background

Postoperative pain remains a clinical priority in bariatric surgery because of its impact on recovery, patient satisfaction, and postoperative complications. However, pain management after bariatric surgery is challenging because of inherent risks, such as respiratory complications, particularly in patients with obstructive sleep apnea (OSA). Current pain management strategies in bariatric surgery remain insufficient, with moderate-to-severe pain reported in 30-50% of patients within 24 hours postoperatively and up to 75% in those using intravenous (IV) patient-controlled analgesia (PCA) after post-anesthesia care unit (PACU) discharge, despite multimodal approaches.

Objectives

To identify perioperative predictors of severe postoperative pain and patient satisfaction after bariatric surgery. This study aimed to characterize the postoperative pain trajectory and guide future interventional trials in bariatric patients.

Methods

In this prospective multicenter observational cohort study, 420 adults undergoing bariatric surgery under general anesthesia were followed for the first 48 hours. Data collected included patient demographics, perioperative predictors of postoperative pain, and postoperative pain outcomes at multiple time points from the PACU through 48 hours postoperatively. Pain intensity was measured using the Numerical Rating Scale (NRS) from 0-10, and severe pain was defined as a pain score of >7/10 within the first 24 hours. Patient satisfaction (0-10) and overall pain relief (0-100) were recorded. Secondary outcomes included other pain outcomes during the first 48 hours after surgery. Multivariate logistic regression was used to identify independent predictors of severe postoperative pain, and linear regression analysis was used to identify predictors of patient satisfaction.

Results

Preliminary findings indicate that 153 of 420 patients (36.4%) experienced severe pain during the first 24 hours. Mean satisfaction was 7.14+1.75, and overall pain relief at 24 hours was 7.14+1.752. Predictors of severe pain included preoperative anxiety, female gender, smoking, preoperative chronic pain, worst postoperative pain, and a greater need for analgesics (all P < 0.05). The strongest predictor of severe pain was uncontrolled early worst pain (regression coefficient = +2.41, OR = 11.13 for worst pain, and P <0.001). The area under the receiver operating characteristic (ROC) curve (AUC) = 0.81 for severe pain, and it had a pseudo‑R² of 0.18. The strongest predictor of high satisfaction was effective pain relief (regression coefficient = +1.2, and P <0.001). Other predictors of higher satisfaction included preoperative patient education, local anesthetic (LA) infiltration, the use of postoperative regional anesthesia (RA), and PCA (OR 0.055-0.056 for RA/PCA). An R² value of 0.855 for satisfaction indicates that 85.5% of the variance in satisfaction scores is explained by the predictors.

Conclusions

In this multicenter cohort, severe postoperative pain remained prevalent after bariatric surgery. Targeting modifiable perioperative predictors, such as preoperative anxiety, preexisting pain, and uncontrolled early postoperative pain, may reduce the incidence of severe pain. Furthermore, adopting multimodal analgesia strategies, including pre-incisional LA infiltration, postoperative RA, PCA, and structured patient education, may improve overall outcomes and enhance patient satisfaction.

## Introduction

Despite improvements in surgical techniques, anesthetic management, and multimodal analgesia strategies, many patients still experience inadequate pain relief, especially on the first postoperative day [1,2]. Clinical studies have shown that approximately 80% of surgical patients report insufficient pain relief after surgery, and 30-50% experience severe pain (pain score >7) within the first 24 hours after surgery [3,4]. Effective management of acute postoperative pain remains crucial for enhancing recovery and ensuring successful rehabilitation [5].

Acute postoperative pain following bariatric surgery is particularly challenging because of its impact on recovery outcomes. Uncontrolled pain disrupts enhanced recovery protocols, delays early mobilization, impairs respiratory function, especially in patients already at risk of pulmonary complications, and increases opioid-related side effects [6]. It also negatively affects patient satisfaction and overall quality of recovery. Uncontrolled pain disrupts enhanced recovery protocols, including early mobilization, oral intake resumption, and reduced complications [1].

Many patients undergoing bariatric procedures present with risk factors that complicate pain management planning, including high body mass index (BMI), obstructive sleep apnea (OSA), and increased sensitivity to opioids [7]. Consequently, pain management goals extend beyond reducing pain scores to include minimizing opioid exposure, facilitating recovery milestones, maintaining patient satisfaction with care, and minimizing the risk of progression to chronic postsurgical pain [7-9].

In bariatric surgery, most available evidence derives from single-center experiences, protocol-driven trials, or studies focused primarily on opioid consumption rather than patient-centered outcomes such as satisfaction or perceived pain relief [10]. Moreover, early postoperative pain is dynamic, and describing its trajectories across clinically relevant time points, such as the post-anesthesia care unit (PACU), ward admission, and subsequent postoperative hours, can reveal when patients are most vulnerable and which interventions appear most protective in routine practice [11,12].

This study is important because severe postoperative pain after bariatric surgery remains common and significantly affects recovery, opioid use, and patient satisfaction. This work aims to identify patients at the highest risk of severe pain and to determine which modifiable perioperative factors are associated with better patient-reported outcomes. Understanding these predictors and evaluating current analgesic practices are essential steps toward generating evidence to optimize pain management protocols in this high-risk population.

Aim of the study

This study aims to identify the key predictors of severe pain and patient satisfaction among patients undergoing bariatric surgery and to characterize postoperative pain trajectories within the first 48 hours. Additionally, it seeks to evaluate the effectiveness of current pain management strategies and to identify targeted interventions that may reduce the incidence and severity of postoperative pain while enhancing patient satisfaction.

## Materials and methods

Study design

This was a prospective, multicenter observational cohort study designed to assess postoperative pain outcomes following bariatric surgery and to identify perioperative predictors of severe pain and patient satisfaction. The study was conducted and reported in accordance with the Strengthening the Reporting of Observational Studies in Epidemiology (STROBE) guidelines [13]. 

Setting

This international multicenter study was conducted across tertiary-care centers in Saudi Arabia (King Khalid University Hospital and King Abdulaziz University Hospital), Egypt (Minia University Hospitals), and the United States (NEMA Research Inc.). Data collection was standardized across all centers using a predefined protocol for assessing pain during the first 48 hours after surgery.

Ethical considerations

The study was part of a quality improvement project aimed at improving postoperative pain outcomes. Ethical approval was obtained from the Institutional Review Board (IRB) of the College of Medicine, King Saud University, Riyadh, Saudi Arabia (Ref. No. 18/0443/IRB). The trial was registered at clinicaltrials.gov (NCT05624502, registration date: January 1, 2019) before patient enrollment. Written Informed consent was obtained from all participants before enrollment in the study.

Participants

A total of 420 adult patients (both males and females) undergoing elective bariatric surgery under general anesthesia (with or without ultrasound-guided fascial plane block) were included. The study data were collected between May 2020 and May 2025.

Inclusion Criteria

The study involved both adult male and female patients aged (≥18 years), with American Society of Anesthesiologists (ASA) physical status II-III, and those who consented to participate in the study. Patients undergoing primary bariatric procedures (e.g., laparoscopic sleeve gastrectomy), with no known allergy to the anesthetic or analgesic medications used in the study. Inclusion requires the availability of complete data for up to 48 hours after surgery.

Exclusion Criteria

Exclusions included patient refusal to participate or inability to complete the study protocol. Patients with significant systemic diseases, significant respiratory problems (e.g., on continuous positive airway pressure (CPAP) or dependent on oxygen therapy, and those who required ICU admission for postoperative care were excluded from the study. Patients undergoing revision of bariatric surgeries, those who developed surgical or anesthetic complications throughout the perioperative course, were excluded from the study. Drug abusers or opioid tolerant patients were also excluded from the study.

Data collection

During the routine preoperative assessment, the anesthesia team explained the anesthetic plan, postoperative pain assessments using the NRS, and the available options for postoperative pain control (e.g., patient-controlled analgesia (PCA) pump and ultrasound-guided fascial plane block). Standardized preoperative education was provided to all patients as part of the institutional quality improvement program. 

Baseline and perioperative variables were collected from the electronic medical record and structured patient assessment forms. Postoperative data were collected by a well-trained member of the acute pain service team on postoperative day one. For patients discharged before 48 hours, follow-up assessments were completed via telecommunication.

Variables and Outcome Measures

**Figure 1:**
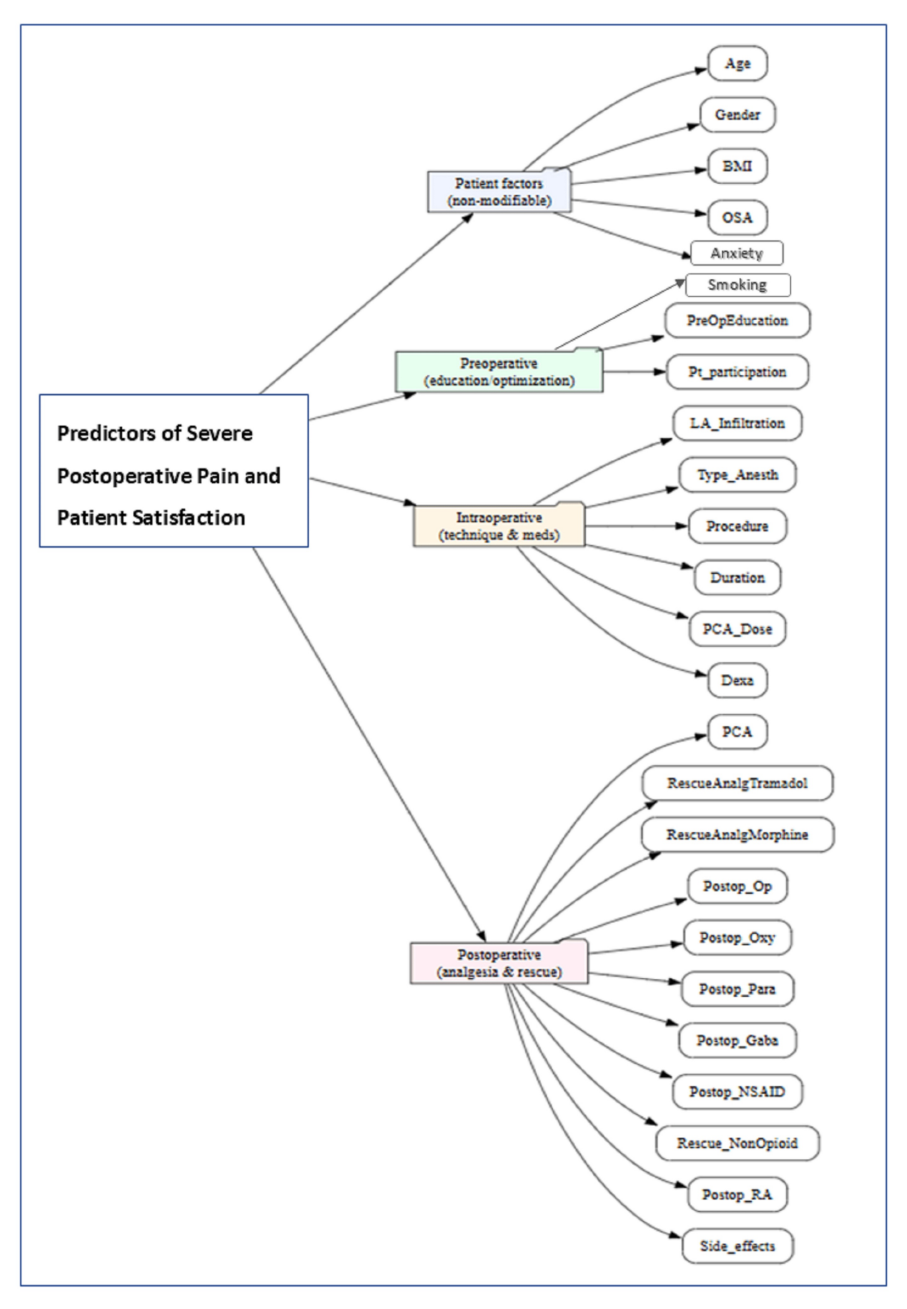
Perioperative predictors of severe pain and patient satisfaction The image was created by the corresponding author. OSA: obstructive sleep apnea, RA: regional anesthesia, LA: local anesthetic, NSAID: nonsteroidal anti-inflammatory drug, PCA: patient-controlled analgesia

The following variables were collected and categorized as patient factors, preoperative, intraoperative, and postoperative factors (Figure 1).

Patient Demographics

This included age (years), gender (male or female), BMI (Kg/m2), smoking status (yes or no), and weight reduction during the last six months.

Preoperative Variables

History of chronic preoperative pain (duration more than three months), preoperative anxiety (yes or no), patient education about the methods of anesthesia, postoperative pain relief, and involvement in shared decision-making.

Intraoperative Factors

This included type of surgery, anesthetic plan, use of pre-incisional local anesthesia (LA) infiltration, intraoperative opioid consumption, and postoperative regional anesthesia (RA; ultrasound-guided transversus abdominis plane (TAP) or erector spinae plane (ESP)), and surgical duration.

All patients received general anesthesia. At the conclusion of surgery and before awakening the patient from anesthesia, an ultrasound-guided fascial plane block (transversus abdominis plane, TAP, or erector spinae plane, ESP was performed in some patients.

Postoperative Variables

Pain outcomes: Pain intensity scores were measured using the NRS from (0 = no pain, to 10 = worst pain) at multiple intervals: immediately in the PACU, at the time of admission to the ward or day surgery unit (DSU), then at one, three, six, 12, 24, and 48 hours postoperatively, as well as before patient’s discharge. For patients discharged earlier, before 48 hours, a follow-up call was made after 48 hours to evaluate the patient's pain and collect the remaining data.

Pain experience: Worst and least pain scores during the first 24 hours, incidence of severe breakthrough pain, total duration of time in severe pain, and overall pain relief (0-100%) were also evaluated.

Analgesic management: All participating centers followed the same predetermined protocol using scheduled simple analgesics (paracetamol 1g IV every six hours, nonsteroidal anti-inflammatory drugs (NSAIDs)/lornoxicam 8mg every eight hours if not contraindicated) plus rescue opioid analgesia. Protocols permitted flexible use of patient-controlled analgesia (PCA) or ultrasound-guided fascial plane blocks (TAP or ESP) based on clinical judgment, reflecting real-world multicenter practice while maintaining core standardization. Postoperative analgesia, RA techniques, patient’s request for more rescue analgesia, and home medications were also recorded.

Patient satisfaction: Rated on a numeric rating scale from 0 to 10.

Possible adverse events: incidence of nausea, vomiting, sedation, or respiratory depression.

Objectives

This multicenter study aimed to identify key predictors of severe postoperative pain and patient satisfaction to enable targeted interventions that reduce the incidence and severity of postoperative pain and improve patient satisfaction after bariatric surgery. Pain intensity was assessed using the NRS (0-10), where 0 = no pain and 10 = worst pain. Pain scores were recorded immediately in the PACU, upon admission to the DSU or ward, and at one, three, six, 12, 24, and 48 hours postoperatively. Patient satisfaction was assessed using a numerical scale from 0 to 10, where 0 = extreme dissatisfaction and 10 = excellent satisfaction. 

Primary Objectives

To evaluate the incidence and perioperative predictors of severe postoperative pain and patient satisfaction during the first 24 hours following bariatric surgery. Severe pain is defined as a pain score ≥7 on an NRS from 0-10 within the first 24 hours. 

Secondary Objectives

To assess additional pain outcomes at different time points, including pain intensity scores at multiple postoperative time points, worst and least pain scores during the first 24 hours, duration of time in severe pain and patient’s request for additional analgesia, and the overall pain relief during the first 24 hours (scored 0-100; 0 = no relief, 100 = excellent relief).

Potential independent perioperative predictors of severe pain included demographic factors (e.g., age and gender), patient-specific factors (e.g., BMI, OSA, and smoking), preoperative factors (e.g., preoperative anxiety, preexisting chronic pain, patient education regarding the anesthesia and analgesia plans, and participation in shared decision-making), and intraoperative clinical factors (e.g., pre-incisional local anesthetic infiltration, duration of surgery, postoperative ultrasound-guided regional analgesia such as ESP or TAP blocks, and PCA). 

Sample size

Based on previous studies, a 1-point difference in pain scores (as measured by the NRS) has been considered clinically significant. Assuming a standard deviation (SD) of 2 points, 80% power, and a two-sided significance level of 0.05, a sample size of 180-200 patients was sufficient to achieve adequate statistical power. To accommodate potential dropouts and exclusions, the target sample size was increased by approximately 10%. As part of this continuous quality improvement project focused on improving postoperative pain outcomes, a total of 420 patients who underwent bariatric surgery were enrolled across multiple centers between May 2021 and May 2025. This larger sample size ensures adequate statistical power for the planned analyses.

Statistical analysis

The data were collected from all patients and analyzed using SPSS version 27 (IBM Corp., Armonk, NY, USA). Continuous variables (e.g., age, BMI, duration of surgery, and pain scores) were summarized as mean ± SD and ranges. Categorical variables (e.g., gender, use of RA, PCA use, and incidence of severe pain) were summarized as counts and percentages (%). Missing data were minimal, and analyses were performed using complete‑case analysis; no imputation was applied.

Differential statistics included the following analyses: repeated-measures ANOVA for pain trajectories over time. An unpaired t-test was used to compare data between two groups. Chi-square was used to compare non-parametric data (e.g., gender, analgesia, or anesthesia techniques). A two-sided P-value < 0.05 was considered statistically significant.

The associations between predictors and outcomes were assessed using correlation analysis. For associations between two continuous, approximately normally distributed variables (e.g., age, BMI, NRS pain scores, satisfaction, pain relief), Pearson’s correlation coefficient was used. For associations involving ordinal or clearly non-normally distributed variables (e.g., ASA class, duration categories where applicable) or when one variable was binary (e.g., presence of OSA, smoking, preoperative anxiety, preoperative chronic pain, severe pain), Spearman’s rank correlation coefficient was used.

Two regression models were performed. Logistic regression analysis was used to identify predictors of severe postoperative pain (NRS ≥7), with odds ratios (ORs) and 95% confidence intervals (CIs). Linear regression analysis was used to identify factors associated with patient satisfaction, including main effects and interaction terms. A two-sided P-value < 0.05 was considered statistically significant for all analyses.

## Results

Patient characteristics

A total of 420 adult male and female patients who underwent bariatric surgery (laparoscopic sleeve gastrectomy) under general anesthesia were included (Table 1 and Appendix 1). The mean age was 35.645+9.77 years (range 18-66), and the mean BMI was 40.79+4.49 kg/m² (range 32-58). Subgroup comparisons between male and female patients to identify sex-based differences in baseline characteristics, pain outcomes, and analgesia utilization. The study included 179 men (42.6%) and 241 women (57.4%). ASA physical status was ASA II in 259 (61.7%), and ASA III in 161 patients (38.3%).

**Table 1:** Baseline Characteristics of the Study Participants BMI = body mass index, OSA = obstructive sleep apnea, ASA American Society of Anesthesiologists, LA = local anesthesia, GA = general anesthesia, RA = regional anesthesia, TAP = transversus abdominis plane, ESP = erector spinae plane, PCA = patient-controlled analgesia. Data expressed as mean (+SD) & Comparison between the groups by an independent t-test (*) Data expressed as (mean + SD) and comparison by Chi-Square test P < 0.05 is significant

Variables	All patients	Male	Female	P
Age /year (mean + SD)	35.64+9.77	37.14+9.93	34.53+9.52	0.007
Gender: N (%)^*^	420	179 (42.6%)	241 (57.4%)	0.002
BMI (kg/m²)	40.79+4.49	42.01+5.32	39.88+3.50	0.001
OSA: N (%)^*^	116 (27.61%)	62 (34.6%)	54 (22.4%)	0.006
Smoking habit: N (%)^*^	119 (28.3%)	72 (40.2%)	47 (19.5%)	<0.001
ASA physical status II: N (%)^*^	259 (61.7%)	97 (54.2%)	162 (67.2%)	<0.001
ASA physical status III: N (%)^*^	161 (38.3%)	82 (45.8%)	79 (32.8%)	
Preop chronic pain: N (%)^*^	139 (33.1%)	63 (35.2%)	76 (31.5%)	0.431
Preop anxiety: N (%)^*^	232 (55.24%)	112 (32.4%)	120 (49.8%)	0.009
Preop weight reduction: N (%)^*^	188 (44.8%)	78 (43.6%)	110 (45.6%)	0.674
Preop education: N (%)^*^	278 (66.2%)	121 (67.6%)	157 (65.1%)	0.599
Participation in decision: N (%)^*^	247 (58.8%)	110 (61.5%)	137 (56.8%)	0.343
Pre-incisional LA: N (%)^*^	304 (72.4%)	125 (69.8%)	179 (74.3%)	0.314
GA: N (%)^*^	260 (61.9%)	108 (60.9%)	152 (63.5%)	0.588
GA + RA: N (%)^*^	160 (38.1%)	71 (39.1%)	89 (36.5%)	
Postop RA: N (%)^*^	160 (38.1%)	71 (16.9%)	89 (21.2%)	0.109
TAP block: N (%)^*^	61 (38.1%)	33 (46.5%)	28 (31.5%)	
ESP block: N (%)^*^	99 (61.9%)	38 (53.5%)	61 (68.5%)	
Duration of surgery (Min)	77.02+13.56	76.396+14.10	77.489+14.03	0.431
Postop PCA: N (%)^*^	222 (52.9%)	85 (47.5%)	137 (56.8%)	0.037
PCA dose /mg (mean + SD)	11.064+12.70	8.810+12.21	12.738+12.82	0.002

Perioperative variables

Preoperative Variables

Preoperative anxiety was reported in 232 patients (55.24%), and preoperative chronic pain (e.g., low back pain and/or osteoarthritis) was reported in 139 patients (33.1%). OSA was reported in 116 patients (27.61%), and smoking habit was reported in 119 patients (28.3%). Preoperative patient education was conducted in 278 patients (66.2%), and patient participation in decision-making was facilitated in 247 patients (58.8%). Additional details are presented in Table 1 and Appendix 1.

Intraoperative Variables

LA infiltration was performed in 304 patients (72.4%), the mean duration of surgery in minutes was (77.02+13.56), type of RA, ESP in 99 patients (61.9%), or TAP block in 61 patients (38.1%), and PCA use in 222 patients (52.9%) (Table 1 and Appendix 2).

Postoperative Pain Outcomes

**Figure 2:**
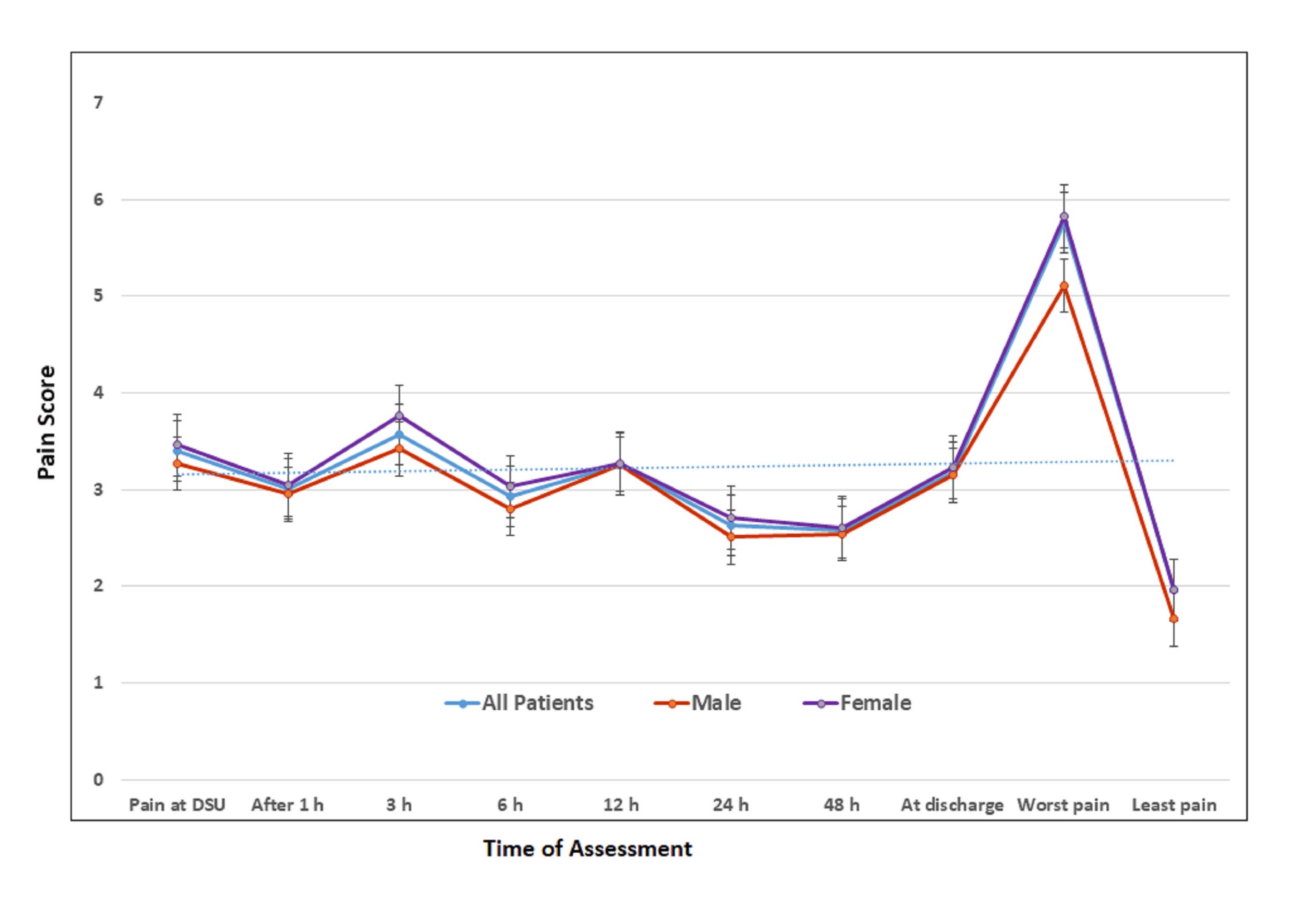
Postoperative pain scores by NRS during the first 48 hours NRS = numerical rating scale, DSU = day surgery unit Data expressed as mean+SD

Pain scores over the first 48 hours are summarized in Table 2, Figure 2, and Appendix 3. The immediate postoperative pain score in the PACU was 2.44+1.83, increasing to 3.4+1.76 on admission to the DSU or ward. The highest pain score within the first 24 hours was 5.76+1.60, and the lowest was 1.96+1.15. Severe pain (NRS >7) during the first 24 hours occurred in 154 patients (36.4%), with a significantly higher incidence in females than in males (P = 0.045). Requests for additional analgesia due to inadequate pain control were reported in 134 patients (31.9%), with comparable frequencies between sexes. The pain trajectory during the first 48 hours showed higher scores in females than in males, although the differences were not significant in most readings.

**Table 2:** Postoperative pain outcomes and satisfaction of the study participants Data expressed as mean (+SD) & Comparison between the groups by an independent t-test (*) Data are expressed as numbers (%) & Comparison by Chi-Square test Severe pain is defined as NRS ≥7 on NRS (0-10). P <0.05 is significant

Variables	All Patients	Male	Female	P
Pain in PACU	2.44+1.83	2.56	2.35	0.179
Pain at DSU/Ward	3.4+1.76	3.27	3.46	0.088
After 1 hour	3.01+1.68	2.95	3.05	0.134
After 3 hours	3.57+1.49	3.42	3.76	0.016
After 6 hours	2.93+1.59	2.8	3.03	0.192
After 12 hours	3.26+1.47	3.26	3.27	0.759
After 24 hours	2.63+1.27	2.51	2.71	0.063
After 48 hours	2.58+1.24	2.54	2.61	0.708
Pain at discharge	3.18+1.32	3.15	3.23	0.488
Worst pain score (in 24 h)	5.76+1.60	5.11	5.83	0.418
Lowest pain score	1.96+1.15	1.66	1.96	0.817
Severe pain^*^	153 (36.4%)	68 (38%)	85 (35.3%)	0.035
Duration of worst pain	35.738+21.881	36.145+23.167	35.435+20.919	0.795
Need analgesia^*^	134 (31.9%)	58 (32.4%)	76 (31.5%)	0.916
% of Pain relief	71.678+14.598	71.145+16.1	72.074+13.395	0.519
Satisfaction	7.14+1.752	7.12+1.84	7.16+1.68	0.768

The overall pain relief at 24 hours was 71.678+14.60 (range 30%-90%), with no significant sex differences. The mean satisfaction score was 7.14+1.75 (range 2-10), and there was no significant difference between male and female patients (P = 0.768) (Table 2, Figure 2, and Appendix 3).

Perioperative Analgesia

Key perioperative analgesic strategies are summarized in Table 2 and Appendices 2 and 3.

Pre-incisional LA infiltration: LA infiltration was performed in 304 patients (72.4%), with the highest use among women.

Postoperative RA (fascial plane block): All included patients (N = 420) received general anesthesia. Postoperative RA in the form of a fascial plane block was performed in 160 patients (38.1%), including a TAP block in 61 of 160 patients (38.1%) and an ESP block in 99 of 160 patients (61.9%). The remaining 260 patients (61.9%) received general anesthesia only without additional postoperative RA.

PCA: Postoperative PCA was used in 222 patients (52.9%), with higher utilization among female patients than among male patients (56.8% vs 47.5%, P = 0.037). The cumulative PCA morphine dose was significantly higher in female patients than in male patients (12.738+12.82 vs 8.810+12.21 mg, P = 0.002).

Other perioperative analgesia: Use of perioperative rescue opioid boluses and regular non-opioid analgesia (paracetamol and NSAIDs if not contraindicated) was comparable between male and female patients.

Correlation analysis

**Figure 3:**
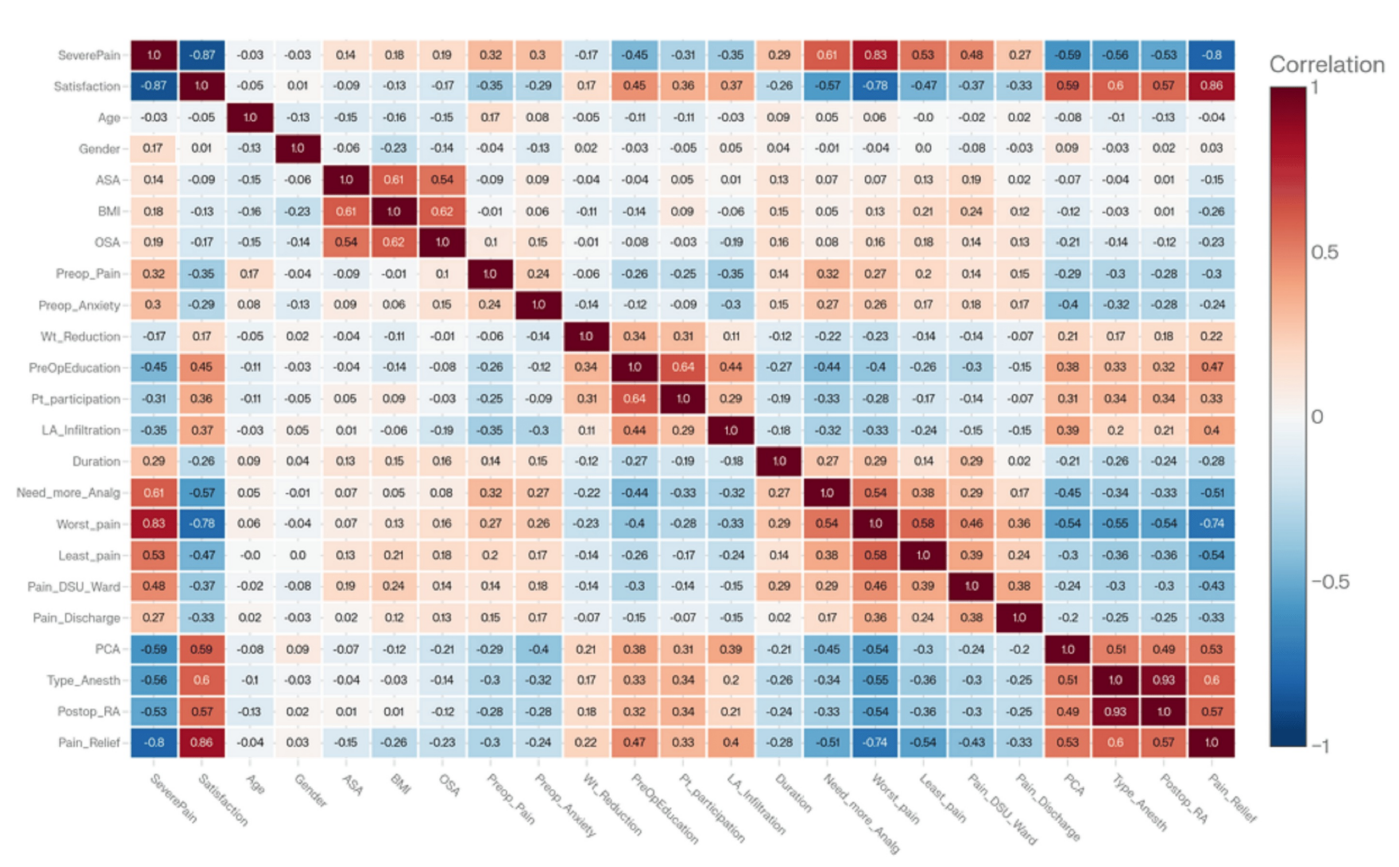
Correlation coefficient heatmap of the perioperative variables ASA American Society of Anesthesiologists, BMI = body mass index, OSA = obstructive sleep apnea, Wt_reduction = weight reduction, PreOpEducation = preoperative education, Pt_participation = patient participation, LA = local anesthesia, RA = regional anesthesia, PCA = patient-controlled analgesia.

The correlation heatmap (Figure 3) illustrated the strength and direction of associations between clinical variables and pain-related outcomes. Correlation analyses of clinical interventions, such as postoperative RA, PCA, and patient education, revealed strong negative correlations with pain outcomes (e.g., severe pain, worst pain, requests for more analgesia), indicating a protective effect. In contrast, preoperative anxiety and early postoperative pain positively correlated with severe postoperative pain, indicating a higher risk. As expected, satisfaction and overall pain relief were strongly and positively correlated (Figure 3).

Strong Positive Correlations

Satisfaction demonstrated the strongest positive correlation with pain relief (r = 0.86, p < 0.001). This reinforces that satisfaction more closely reflects overall perceived pain relief than absolute pain scores. Correlation analyses between satisfaction and RA (r = 0.60, p < 0.0) and PCA (r = 0.59, p < 0.01) confirm that adding RA and/or PCA improved pain outcomes and patient satisfaction.

Similarly, severe pain was strongly correlated with the worst pain (r = 0.83, p <0.001) and the need for additional analgesia (r = 0.61, p <0.001). This confirms that peak pain intensity and subsequent requests for rescue analgesia are key determinants of severe pain outcomes.

Strong Negative Correlations

Satisfaction showed a strong negative correlation with severe pain (r = -0.87, p <0.001) and worst pain (r = -0.78, p <0.001), indicating that high pain scores are nearly incompatible with high satisfaction, making high pain scores an important risk factor for low patient satisfaction.

Severe pain, on the other hand, showed a strong negative correlation with PCA (r = -0.59, p <0.001) and postoperative RA (r = -0.53, p <0.001), providing strong statistical evidence of the efficacy of such interventions in reducing severe pain.

Moderate Positive Correlations

Satisfaction showed mild to moderate positive correlations with preoperative education (r = +045), pre-incisional LA infiltration (r = +0.31), and patient participation in decision-making (r = +0.31). 

Conversely, severe pain showed mild to moderate positive correlations with smoking (r = 0.35), preoperative chronic pain (r = 0.32), preoperative anxiety (r = 0.30), and female gender (r = 0.28).

Moderate Negative Correlation

Satisfaction also showed moderate negative correlations with preoperative pain (r = -0.35), preoperative anxiety (r = -0.29), female gender (r = -0.25), and smoking (r = -0.20). Similarly, severe pain showed moderate negative correlations with preoperative education (r = -0.45), LA infiltration (r = -0.35), and patient participation in the decision (r = -0.31).

Mild (Weak) Positive Correlation

Satisfaction and preoperative weight reduction showed weak positive correlations (r = 0.17). Similarly, severe pain showed weak positive correlations with OSA (r = 0.19), BMI (r = 0.18), female gender (r = 0.17), and ASA (r = 0.14).

Mild (Weak) Negative Correlation

Satisfaction showed a weak negative correlation with the duration of surgery (r = -0.26), OSA (r = -0.17), BMI (r = -0.13), and ASA physical status (r = -0.09). Severe pain also showed a weak negative correlation with weight reduction (r = -0.17).

Regression analysis

Linear Regression for Predictors of Satisfaction

**Figure 4:**
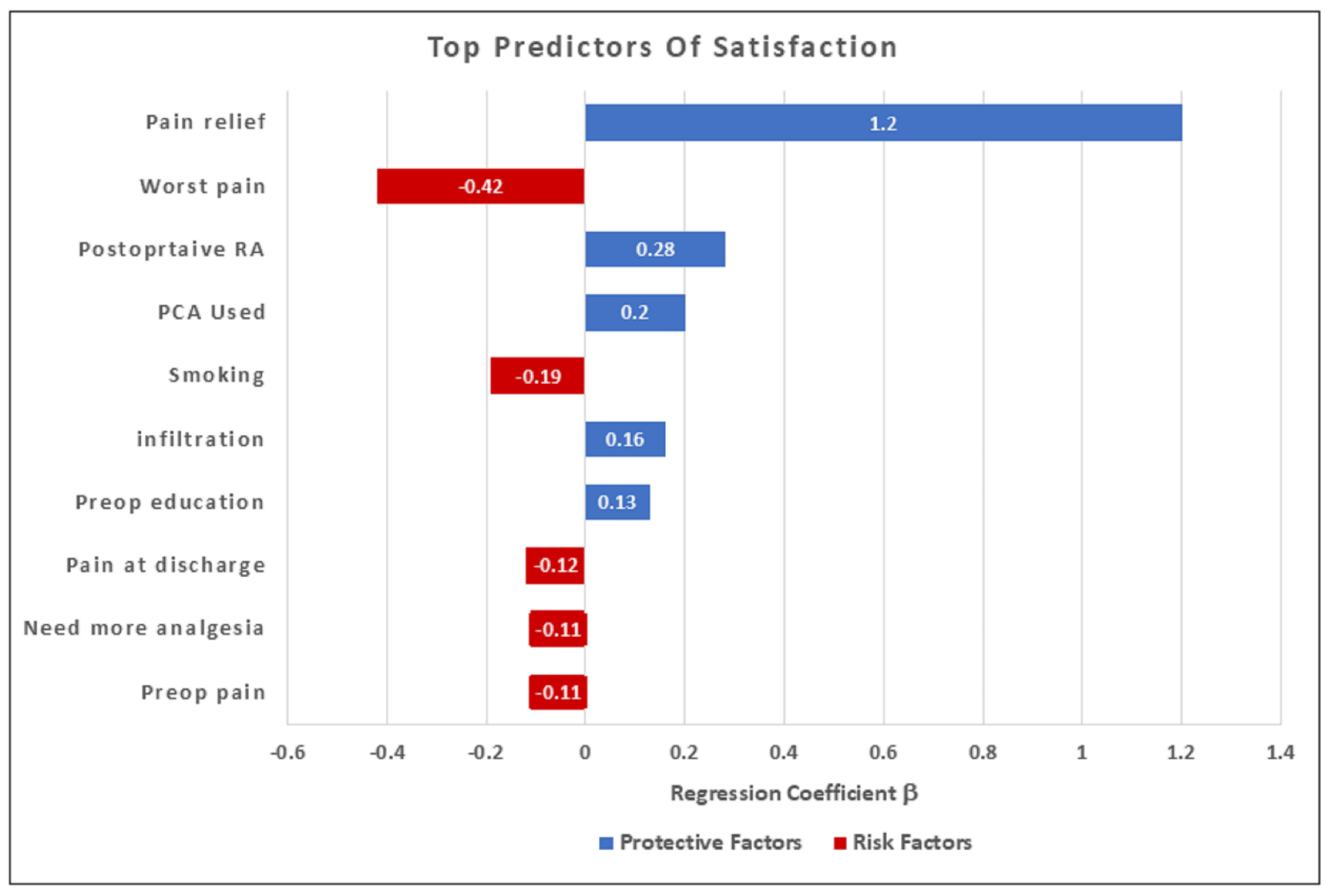
Linear regression analysis of the top ten predictors of patient satisfaction

The linear regression model for satisfaction showed an R2 value of 0.855. This result indicates that 85.5% of the variance in satisfaction scores could be explained by these predictors. Multivariate linear regression (Table 3 and Figure 4) for patient satisfaction showed that overall pain relief was the strongest independent predictor of high satisfaction. Higher satisfaction was also independently associated with postoperative RA (β = +0.28, P<0.001), PCA use (β = +0.20, P = 0.002), pre-incisional LA infiltration (β = +0.16, P = 0.015), and preoperative patient education (β = +0.13, P = 0.031). These positive coefficients indicate that as these variables increase, patient satisfaction also increases; accordingly, they act as protective factors. In contrast, negative coefficients, such as higher worst pain (β = −0.42, P <0.001), smoking (β = −0.19, P = 0.008), and higher pain at discharge (β = −0.12, P = 0.047), were associated with lower satisfaction; they act as risk factors. The coefficients for patients’ need for more analgesia (β = −0.11, P = 0.068) and preoperative chronic pain (β = −0.11, P = 0.069) showed an insignificant negative association.

**Table 3:** Linear Regression Analysis for Predictors of Patient Satisfaction RA = Regional Anesthesia LA = Local Anesthesia A positive coefficient indicates a protective factor. A negative coefficient indicates a risk factor. P-value <0.05 is considered significant. R2 = 0.855 (Interpretations: 85.5% of variation in satisfaction scores explained by predictors)

Variable	Coefficient (β)	Direction	95% CI		P-Value	Interpretation
			Lower	Upper		
Pain Relief	+1.20	Positive	0.044	0.061	<0.001	Strongest predictor of high satisfaction
Worst Pain (in 24 h)	-0.42	Negative	-0.169	-0.004	<0.001	Strongest predictor of low satisfaction
Postop RA	+0.28	Positive	-0.119	0.266	<0.001	Increases satisfaction
PCA Used	+0.20	Positive	-0.276	0.198	0.002	Increases satisfaction
Smoking	-0.19	Negative	-0.350	-0.029	0.008	Decreases satisfaction
LA Infiltration	+0.16	Positive	-0.302	0.073	0.015	Increases satisfaction
Preop Education	+0.13	Positive	-0.243	0.190	0.031	Increases satisfaction
Pain at Discharge	-0.12	Negative	-0.196	-0.014	0.047	Decreases satisfaction
Need More Analgesics	-0.11	Negative	-0.315	0.066	0.068	Decreases satisfaction
Preop Pain	-0.11	Negative	-0.248	0.75	0.069	Decreases satisfaction

The linear regression model for satisfaction had an R² of 0.855 and an adjusted R² of 0.848, indicating that approximately most of the data (85%) of the variance in satisfaction scores was explained by the included predictors, with residual diagnostics supporting the assumptions of linear regression, which is consistent with the strong correlations between satisfaction and pain relief (r ≈ 0.86) and severe pain (r ≈ −0.87) (Table 3 and Figure 4).

Logistic Regression for Prediction of Severe Pain

**Figure 5:**
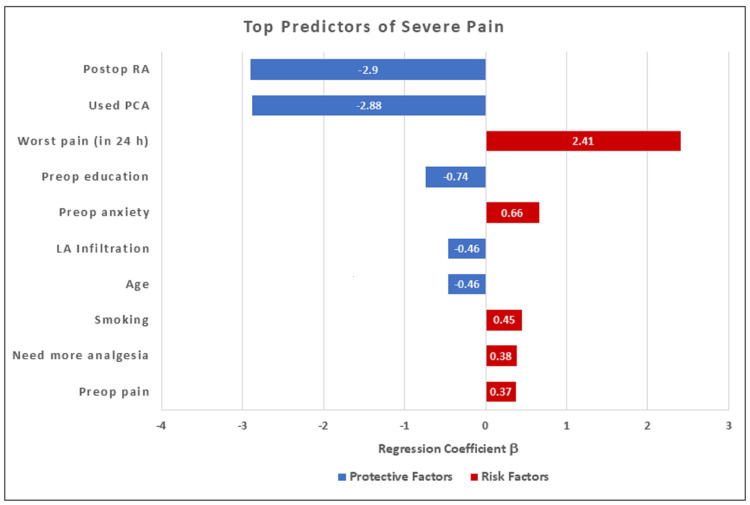
Logistic regression analysis of the top ten predictors of severe pain

Logistic regression analysis (Table 4 and Figure 5) reported the coefficients (*B*) and calculated ORs for the top predictors of severe pain.

**Table 4:** Logistic regression analysis for predictors of severe pain PCA = Patient Controlled Analgesia, LA = Local Anesthesia, Postop RA = Postoperative Regional Anesthesia. OR<1 (Protective): Reduced risk of severe pain OR>1 (Risk): Increased risk of severe pain Pseudo‑R² = 0.18, Area under the receiver operating characteristic curve (AUC) = 0.81

Variable	Coefficient (B)	Odds Ratio (OR)	P-Value	Interpretation
Postop RA	-2.90	0.055	<0.001	Strong reduction in risk of severe pain
PCA Used	-2.88	0.056	<0.001	Strong reduction in risk of severe pain
Worst Pain (in 24 h)	+2.41	11.13	<0.001	High worst pain scores strongly predict severe pain
Pre-Op Education	-0.74	0.477	<0.001	Reduces odds by 52%
Pre-Op Anxiety	+0.66	1.93	<0.001	Nearly doubles the odds of severe pain
LA Infiltration	-0.46	0.631	0.002	Reduces odds by 37%
Age	-0.46	0.631	0.003	Older age reduces odds of severe pain
Smoking	+0.45	1.57	0.004	Increases odds by 57%
Need More Analgesia	+0.38	1.46	0.018	Increases odds by 46%
Preop Pain	+0.37	1.45	0.021	Increases odds by 45%

Postoperative RA and PCA use were associated with a significant reduction in the odds of severe pain (B = −2.90, OR = 0.055, P <0.001) and (B = −2.88, OR = 0.056, P <0.001), respectively, and were among the acceptable protective factors. Preoperative education (B = −0.74, OR = 0.48, P <0.001), LA infiltration (B = −0.46, OR = 0.63, P = 0.002), and older age (B = −0.46, OR = 0.63, P = 0.003) were also independently associated with lower odds of severe pain. Conversely, higher worst postoperative pain scores and preoperative anxiety were strongly associated with severe pain (B = +2.41, OR = 11.13, P<0.001) and (B = +0.66, OR = 1.93, P <0.001), respectively. Additional risk factors associated with severe pain were smoking habit (B = +0.45, OR = 1.57, P = 0.004), patients' need for more analgesia (B = +0.38, OR = 1.46, P = 0.018), and preoperative chronic pain (B = +0.37, OR = 1.45, P = 0.021).

The logistic regression model showed acceptable overall fit, with a pseudo-R² of 0.18, indicating that the included predictors explained a meaningful proportion of the variation in severe postoperative pain. Discriminative ability was good, with an area under the receiver operating characteristic (ROC) curve (AUC) of 0.81, meaning that in about 81% of patient pairs (one with severe pain and one without), the model correctly assigned a higher predicted risk to the patient who actually experienced severe pain (Table 4 and Figure 5).

## Discussion

This multicenter study demonstrates that severe postoperative pain remains common after bariatric surgery (36.4% of patients), despite widespread use of multimodal strategies. Severe pain is mainly linked to the highest pain intensity, preoperative anxiety, smoking, preoperative chronic pain, and the need for rescue analgesia, along with patient-specific factors such as higher BMI, OSA, and female sex. Postoperative RA, PCA, LA infiltration, and preoperative education were protective and strongly associated with higher satisfaction. These findings support integrating multimodal analgesia and patient-specific strategies into enhanced recovery pathways [14].

Because this was an observational cohort, the identified predictors should be interpreted as hypothesis-generating signals rather than proof of causality, and they are intended to guide the design of future interventional studies in bariatric postoperative pain management.

Predictors of severe pain and patient satisfaction

Severe Postoperative Pain

The incidence of severe postoperative pain (36.4%) confirms that acute pain remains a major problem following bariatric surgery. This aligns with other research reporting that the incidence of severe pain ranged from 30% to 50% [4,5]. The strong association between severe pain, worst pain, early postoperative pain, and requests for more analgesia supports the concept that early, prophylactic, and preemptive management of postoperative pain is crucial to prevent escalation over the first 24 hours [14].

The beneficial effects of these interventions, such as RA (e.g., LA infiltration, TAP, or ESP) and PCA, observed in this study were confirmed by several trials that have shown reduced severe pain, lower opioid consumption, and increased satisfaction [4,15].

The beneficial effect of fascial plane blocks (e.g., ESP and TAP blocks) in the current cohort showed no significant differences between the two techniques. This is consistent with several clinical studies. A randomized controlled trial reported lower 24-hour pain scores and a longer time to the first analgesia request with ESP compared with TAP, although opioid consumption and complications were similar [1,16,17]. At the same time, a meta-analysis indicated that ESP may not always confer a substantial additional benefit over TAP across all procedures, and the effect can vary depending on the block technique, timing, local anesthetic dose, and operator expertise, which may explain the inconsistent findings in some studies [18]. A similar study compared ESP vs TAP in bariatric surgery showed that Key ESP was superior to TAP for pain control from six to 12 hours post‑op; similar safety profiles [19]. While the effect of the ESP block was significant when compared to placebo (no block) and revealed reduced intraoperative and postoperative opioid consumption in patients undergoing laparoscopic sleeve gastrectomy, with better early pain scores in the ESP group [20].

In contrast to these findings, reported inconsistent findings or smaller benefits from the fascial plan blocks after laparoscopic sleeve gastrectomy and other abdominal surgeries. This may be attributed to heterogeneity in block technique (plane, dose, and timing), operator expertise, and the baseline assessment of analgesia [21].

Other preoperative predictors of severe pain, such as anxiety, smoking, and preoperative chronic pain, emerged as important risk factors, consistent with prior work identifying psychological stress, smoking, and central sensitization as predictors of perioperative pain and increased opioid consumption [22,23].

A recent meta-analysis demonstrates that patients who smoke have significantly higher postoperative opioid requirements (around 33.7% more at 24h) and higher pain scores compared to nonsmokers. These findings highlight the need to consider smoking status when developing postoperative pain management strategies [16].

Preoperative anxiety is associated with higher pain scores and severe postoperative pain in many studies and across different surgical procedures, rather than specifically in bariatric surgery.

Smoking is linked to delayed recovery and impaired tissue healing, increased opioid use, and increased comorbidities among those in the bariatric and high-risk populations. Chronic pain leads to central sensitization and increased opioid consumption. Other patient-specific risk factors, such as female gender, BMI, and OSA, have also been linked to higher pain intensity and more challenging pain control after bariatric surgery [9,24].

Patient Satisfaction

Overall satisfaction in this study (mean 7.14/10) ranged from moderate to excellent. It was strongly correlated with and predicted by pain relief (β = +1.20), rather than by pain scores alone. This pattern aligns with clinical studies showing that satisfaction reflects effective postoperative pain management and the timeliness of certain interventions (e.g., pre-incisional LA infiltration, PCA, and fascial plan blocks), as well as proper communication (e.g., patient education and participation in the decision) [25,26]. These interventions positively impacted satisfaction in this cohort, confirming that multimodal, proactive analgesia and patient engagement in decision-making were associated with higher satisfaction.

Multimodal Analgesia

Current perioperative and bariatric anesthesia guidelines emphasize multimodal analgesia protocols because of the known drawbacks of unrelieved pain and the potential risks of high-dose opioid use alone, aiming to balance pain control and safety [27,28]. In this study, multimodal strategies incorporating pre-incisional LA infiltration, RA, PCA (ESP/TAP block), opioid boluses, and non-opioid analgesia (paracetamol and NSAIDs) were widely employed, and this was associated with improved pain outcomes and higher satisfaction. Multimodal analgesia is associated with improved overall pain outcomes and satisfaction. In this study, PCA use was associated with a lower incidence of severe pain and higher satisfaction. These findings are consistent with reports that structured multimodal regimens and educational programs reduce opioid consumption, improve pain outcomes, and enhance recovery after bariatric surgery [29]. This is also confirmed by another study that used PCA in addition to other modalities such as RA and non-opioid analgesia as part of multimodal analgesia. This protocol is based on the assumption that bariatric patients are vulnerable to under-treatment of pain due to concerns about respiratory depression [29-31].

In contrast, a prior study showed a high incidence of moderate to severe pain in patients who underwent bariatric surgery and received PCA for postoperative pain relief [24]. Another study showed that PCA did not invariably reduce pain scores compared with nurse-administered on-demand opioid boluses [27]. This assumption may be explained by the lack of preoperative education on how to use the PCA properly, or when concerns about the risk of respiratory depression limit adequate dosing in high-risk patients, such as those with obesity. 

The relatively weak association between severe pain and BMI or OSA in this cohort aligns with findings from other bariatric studies, where variability in patient characteristics, surgical technique, comorbidities, the extent of preoperative predictors, and psychological factors may modulate the impact of obesity-related risk factors [21,32]. The minimal effect of age showed a very weak correlation with severe pain and satisfaction, which can be explained by the narrow age distribution in the bariatric population, in which most patients fall within a relatively young to middle-aged group. In this study, around 90% are between 20 and 38 years old. This is consistent with the findings of the multivariate analysis that revealed no significant differences in age or sex between those with moderate to severe pain versus patients with no to mild pain postoperatively [24,33]. The pain trajectories and identified predictors form a practical risk‑stratification framework that can be tested in cluster randomized trials of standardized multimodal pathways.

Clinical implication

Minimizing Severe Pain

This study highlighted that severe pain after bariatric surgery is a multidimensional experience including nociceptive input, psychological factors (anxiety), behavioral factors (smoking), communication-related factors (patient education and shared decision-making) and pre-existing chronic pain. Many of these factors are modifiable and represent important targets for improving the quality of postoperative pain outcomes in bariatric surgery. 

Based on the results of this study, any quality improvement plan could therefore include screening and optimization of modifiable factors (e.g., anxiety, pre-existing chronic pain, smoking, preoperative education about the postoperative pain and the analgesic options). The protocol also should emphasize on the use of structured multimodal analgesia. This can be achieved by using schedules analgesia, LA infiltration, fascial plane block, and PCA when feasible. Prevention of early worst pain is the key predictor and may therefore represent early warning signals for patients at risk of slow recovery or chronic postsurgical pain, and justify targeted preventive strategies. Screening and modifying preexisting predictors of severe pain may mitigate risk development [1].

Regression analysis of this cohort revealed that satisfaction depends mainly on effective pain relief rather than on pain intensity alone. To enhance satisfaction, several interventions must be implemented: first, emphasize on improved communications (e.g., preoperative education and patient involvement in decision-making). Second, use available tools to enhance pain relief, such as LA infiltration, RA, and PCA. Third, provide rapid response to early postoperative pain and pain at discharge [2]. These measures are consistent with ERAS principles and are likely to enhance patient satisfaction.

Based on our findings, we recommend preoperative screening and management of anxiety (OR 1.93) and standardized patient education (OR 0.48, P<0.001), universal intraoperative pre-incisional local anesthetic infiltration (OR 0.63, P=0.002), routine postoperative regional anesthesia (OR 0.055, P<0.001) with patient-controlled analgesia protocols, and risk stratification prioritizing female patients, those with chronic pain, and smokers. These evidence-based interventions target the identified 36.4% severe pain incidence and align with enhanced recovery after surgery (ERAS) principles for bariatric surgery.

To enhance clinical impact, future quality improvement work in bariatric surgery could address anxiety, smoking habits, and preexisting pain management, combine analgesic interventions, and monitor pain relief and satisfaction as key process and outcome indicators.

Strengths and limitations

Strengths of the Study

This study has several notable strengths. The study design: prospective, multicenter cohort design, with inclusion of patients from multiple tertiary-care centers, enhances external validity and generalizability compared with single-center studies. ​Furthermore, the large sample size, which includes 420 bariatric surgery patients, provides stronger precision for estimating the incidence of severe pain, supports multivariable modeling, and increases the study's power.​

The study emphasizes patient-centered outcomes (e.g., trajectory, relief, and satisfaction). The multidimensional pain assessment, as the pain was measured repeatedly from PACU through 48 hours, enabled a clinically meaningful pain trajectory rather than a one-time snapshot. The pain assessment was not limited to the NRS, but other dimensions were measured (e.g., worst pain, least pain, pain at admission to PACU, DSU/ward, and at discharge, request for more analgesia, perceived pain relief, and patient satisfaction with the pain management). This comprehensive approach makes the assessment powerful, reasonable, and more patient-centered.

Bariatric-relevant predictors: The analysis included patient-specific factors commonly observed in bariatric populations, such as BMI, OSA, anxiety, and chronic pain conditions often associated with obesity (e.g., low back pain and osteoarthritis), as well as smoking status. Incorporating these variables enhances the clinical relevance of the findings for this population group.

Finally, by including centers from the underrepresented Middle East centers, such as Saudi Arabia and Egypt, the study adds evidence from a region that is underrepresented in much of the perioperative pain literature despite a high prevalence of obesity and increased volumes of bariatric surgery.

Limitations of the Study

Several limitations should be acknowledged. The observational design precludes causal inference regarding the effects of some interventions (e.g., RA, fascial plan block, and PCA) on outcomes. Overall pain scores and satisfaction are assessed using self-reported measures. These tools may be influenced by culture, expectations, and communication skills. Additionally, the follow-up period was limited to 48 hours postoperatively, and in some DSU patients, follow-up was completed by telephone after patient discharge. As a result, the study could not address subacute or intermediate outcomes (e.g., functional recovery or transition to chronic pain).

Recommendations and future directions

Routine preoperative screening and optimization of modifiable risk factors, including anxiety, chronic pain, smoking, high BMI, and OSA, should be integrated into bariatric perioperative pathways. Consider RA, local infiltration, and PCA for bariatric patients as part of multimodal analgesia where feasible. Such trials could evaluate whether systematically involving these foundations within ERAS-based bariatric protocols reduces severe pain, opioid exposure, and dissatisfaction beyond that of current standard care.

Tailor analgesia to the risk profile, including BMI, OSA, and smoking habits, and monitor pain relief and satisfaction as quality metrics.

Future research is needed to validate predictive models across diverse populations. Integrating psychological screening tools (e.g., anxiety and pain expectations) into large prospective databases could refine risk prediction models. Extending follow-up beyond 48 hours would clarify whether early pain prevention could reduce chronic postsurgical pain or improve quality-of-life outcomes. Risk-based protocols are needed in resource-limited settings, particularly within Middle Eastern health systems, where bariatric procedures are common, but data remain limited.

## Conclusions

Severe postoperative pain remains common after bariatric surgery, affecting more than one-third of patients despite multimodal analgesia strategies. Consistency identified predictors include poorly controlled early or worst pain, preoperative anxiety, chronic pain, higher BMI, and limited use of regional techniques. Patient satisfaction was strongly linked to effective pain relief.

Targeted multimodal analgesia, such as pre-incisional LA infiltration, postoperative RA, PCA, and non-opioid analgesia, together with preoperative education and patient participation in decision-making, are crucial to achieving effective pain control and improving outcomes.

These findings support a risk-stratified, patient-centered approach to perioperative pain management in bariatric surgery, in which the systematic use of multimodal regional techniques, proactive control of peak pain, and targeted optimization of high-risk patients are central to reducing severe pain and enhancing satisfaction.

## Figures and Tables

**Figure 6:**
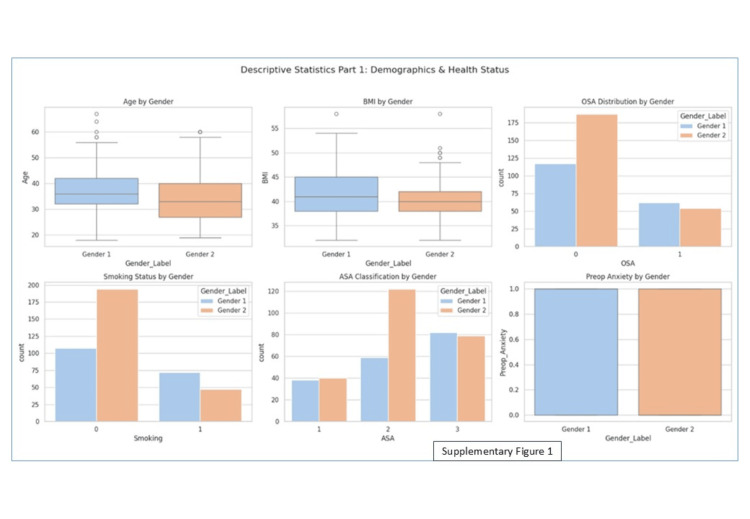
Descriptive Analysis: Demographics and Health Status

**Figure 7:**
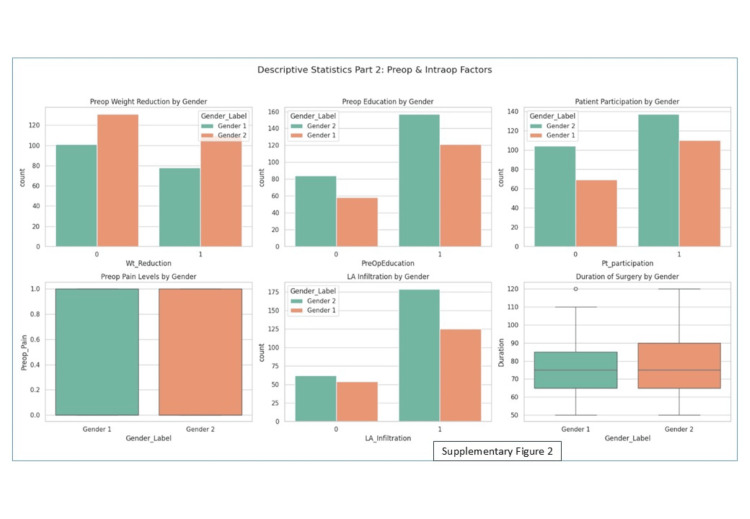
Descriptive Analysis: Pre- and Intra-operative Factors

**Figure 8:**
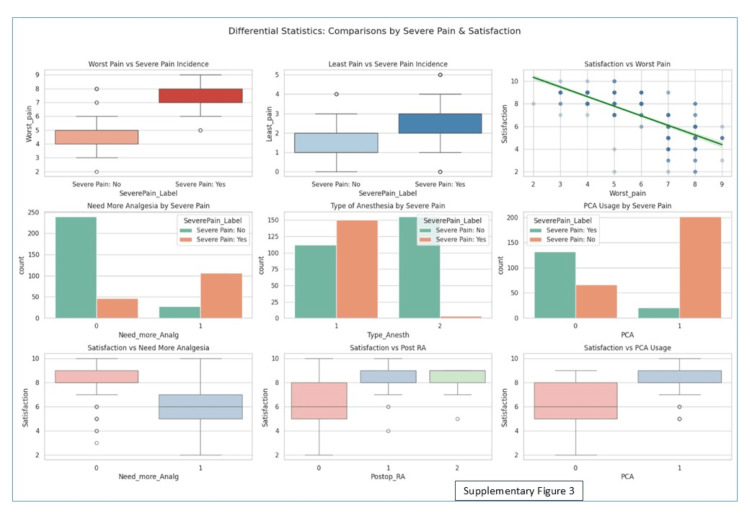
Comparison of Pain Outcomes and Satisfaction

## Appendices

Appendix 1 

Appendix 2  

Appendix 3  
